# Plasma glial fibrillary acidic protein as a biomarker of acute focal brain injury after high-intensity focused ultrasound thalamotomy

**DOI:** 10.1093/braincomms/fcaf054

**Published:** 2025-03-03

**Authors:** Nil Saez-Calveras, Alexander Asturias, James Yu, Barbara Stopschinski, Jaime Vaquer-Alicea, Padraig O'Suilleabhain, Lauren McKenzie, Jeniz Viera, Marc I Diamond, Bhavya R Shah

**Affiliations:** Center for Alzheimer’s and Neurodegenerative Diseases, Peter O’Donnell Jr. Brain Institute, University of Texas Southwestern Medical Center, Dallas, TX 75390, USA; Department of Neurology, University of Texas Southwestern Medical Center, Dallas, TX 75235, USA; Parkland Memorial Hospital, Dallas, TX 75235, USA; Department of Radiology, University of Texas Southwestern Medical Center, Dallas, TX 75390, USA; Transcranial Focused Ultrasound Lab and Program, Department of Radiology, University of Texas Southwestern Medical Center, Dallas, TX 75390, USA; Department of Radiology, University of Texas Southwestern Medical Center, Dallas, TX 75390, USA; Transcranial Focused Ultrasound Lab and Program, Department of Radiology, University of Texas Southwestern Medical Center, Dallas, TX 75390, USA; Center for Alzheimer’s and Neurodegenerative Diseases, Peter O’Donnell Jr. Brain Institute, University of Texas Southwestern Medical Center, Dallas, TX 75390, USA; Department of Neurology, University of Texas Southwestern Medical Center, Dallas, TX 75235, USA; Center for Alzheimer’s and Neurodegenerative Diseases, Peter O’Donnell Jr. Brain Institute, University of Texas Southwestern Medical Center, Dallas, TX 75390, USA; Department of Neurology, University of Texas Southwestern Medical Center, Dallas, TX 75235, USA; Department of Radiology, University of Texas Southwestern Medical Center, Dallas, TX 75390, USA; Transcranial Focused Ultrasound Lab and Program, Department of Radiology, University of Texas Southwestern Medical Center, Dallas, TX 75390, USA; Department of Radiology, University of Texas Southwestern Medical Center, Dallas, TX 75390, USA; Transcranial Focused Ultrasound Lab and Program, Department of Radiology, University of Texas Southwestern Medical Center, Dallas, TX 75390, USA; Center for Alzheimer’s and Neurodegenerative Diseases, Peter O’Donnell Jr. Brain Institute, University of Texas Southwestern Medical Center, Dallas, TX 75390, USA; Department of Neurology, University of Texas Southwestern Medical Center, Dallas, TX 75235, USA; Center for Alzheimer’s and Neurodegenerative Diseases, Peter O’Donnell Jr. Brain Institute, University of Texas Southwestern Medical Center, Dallas, TX 75390, USA; Department of Radiology, University of Texas Southwestern Medical Center, Dallas, TX 75390, USA; Transcranial Focused Ultrasound Lab and Program, Department of Radiology, University of Texas Southwestern Medical Center, Dallas, TX 75390, USA; Department of Neurological Surgery, University of Texas Southwestern, Dallas, TX 75390, USA

**Keywords:** thalamotomy, GFAP, stroke, biomarkers, HIFU

## Abstract

The validation of brain injury biomarkers has encountered challenges such as the absence of pre-insult measurements, variability in injury timing and location, and inter-individual differences. In this study, we addressed these limitations by using magnetic resonance-guided high-intensity focused ultrasound (MRgHIFU) thalamotomy to assess plasma biomarker changes after an acute focal brain injury. This prospective study included 30 essential tremor and tremor-dominant Parkinson’s disease patients undergoing MRgHIFU thalamotomy at a single academic institution. Blood samples were collected at three specific time points: pre-procedure, 1-h post-procedure, and 48 h post-procedure. Plasma levels of glial fibrillary acidic protein (GFAP), neurofilament light chain (NfL), amyloid beta (Aβ40 and Aβ42) and phosphorylated tau 181 (pTau-181) were measured using the quanterix single molecule arrays assay. GFAP levels were significantly increased at 48 h post-MRgHIFU in all patients with a thalamotomy lesion. GFAP levels at 48 h were highly sensitive (89.7%) and specific (96.6%) in detecting the presence of a lesion with a cut-off value of 216.2 pg/ml. NfL, Aβ40 and Aβ42, also showed statistically significant increases post-procedure but were less robust than GFAP. No changes were observed in pTau-181 levels post-MRgHIFU. Plasma GFAP has shown great promise as a sensitive and reliable biomarker for detecting acute brain injury after MRgHIFU thalamotomy. Its significant elevation following the procedure highlights its potential as a diagnostic tool for acute focal brain injuries, such as stroke. Further studies with additional time points are essential to validate the injury cut-off identified in this study and to assess its broader clinical utility for early detection of focal brain lesions.

## Teaser

Saez-Calveras *et al*. report that high-intensity focused ultrasound thalamotomy leads to an acute increase in glial fibrillary acidic protein levels within 48 h after injury. This significant elevation suggests the potential for this biomarker as a diagnostic tool for acute focal brain injuries.

## Graphical Abstract

**Graphical Abstract:**
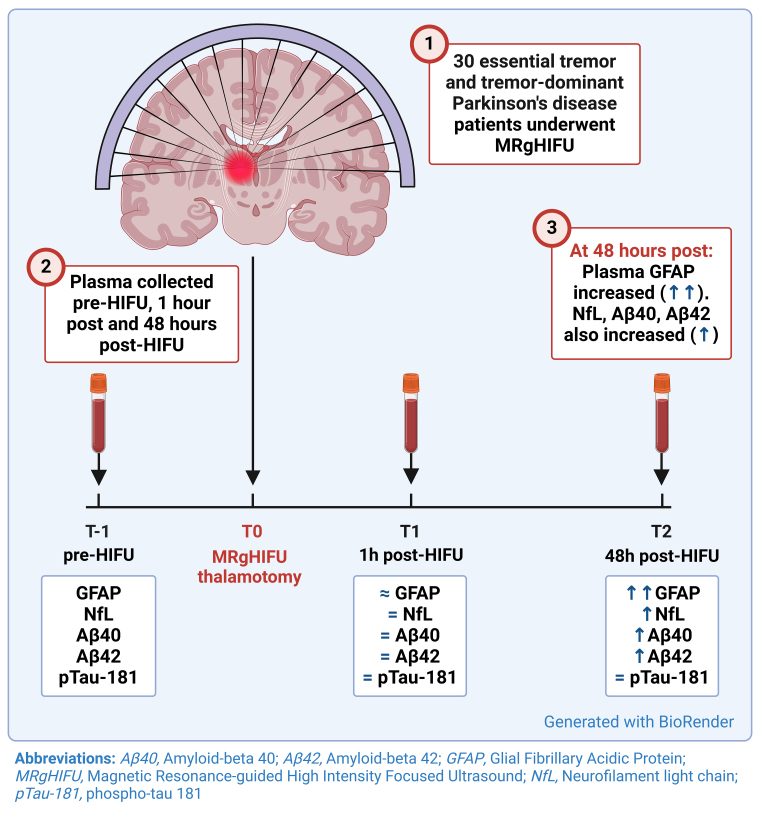

## Introduction

Plasma biomarkers of neurological injury offer significant advantages in clinical practice, including low cost, rapid turnaround time and broad accessibility. Together these factors can substantially impact time to clinical decision making and treatment.^1^ This is particularly relevant in the context of acute stroke, where timely and accurate diagnosis is critical, and no blood-based biomarkers are currently available in clinical practice.^2^ The development and validation of reliable acute brain injury biomarkers could facilitate diagnosis and guide treatment decisions.

Circulating levels of glial fibrillary acidic protein (GFAP),^3^ and neurofilament light chain (NfL),^4^ have emerged as promising biomarkers of acute and subacute brain injury.^5,6^ GFAP is an intermediate filament-III protein primarily expressed in astrocytes, which serves as a marker of astroglial activation and injury.^7^ Meanwhile, NfL is a neuron-specific cytoskeletal component that reflects axonal damage.^8^ Circulating amyloid-β peptides, which are cleavage products of the amyloid precursor protein, and phosphorylated tau species, which are modified forms of the microtubule-associated protein tau, have been proposed as markers of neurodegeneration.^9-12^ However, they can also increase in acute stroke^13^ and traumatic brain injury (TBI).^14^ Identifying the most reliable and consistent biomarkers of brain injury faces a multitude of challenges. These include the lack of pre-insult biomarker measurements,^6,15^ difficulties in accurately determining the timing of injury,^16^ and the variability arising from inter-individual differences such as comorbidities, and peripheral tissue sources of these biomarkers.^17,18^ Addressing these challenges is essential to validate these plasma biomarkers for use in clinical practice.

Magnetic resonance-guided high-intensity focused ultrasound (MRgHIFU) thalamotomy represents a unique opportunity in addressing these limitations. As an FDA-approved, incisionless therapy for essential tremor (ET)^19^ and tremor-dominant Parkinson’s disease (TDPD), this treatment ablates the dentatorubrothalamic tract (DRTT)/ventral intermediate nucleus (VIM) of the thalamus providing real-time tremor relief.^20^ MRgHIFU ablation also results in temporary disruption of the blood–brain-barrier (BBB).^21,22^ We have an established MRgHIFU program where ET and PD patients routinely undergo MRgHIFU ablation using a precision-imaging based approach named four tract tractography.^20,23^ This approach improves clinical outcomes and reduces adverse effects by using patient-specific tractography instead of stereotactic coordinates.^20^ At the same time, MRgHIFU with four tract tracrography provides a precise and consistent ablation of a circumscribed anatomic area in the DRTT/VIM of the thalamus across different patients, with minimal target heterogeneity.^20^

By utilizing the controlled timeline and spatial precision of MRgHIFU thalamotomy, we can closely examine the relationship between specific plasma biomarkers and a brain injury that closely resembles that seen in thalamic stroke. This approach eliminates the biases introduced by time variability and inter-individual differences that complicate other biomarker studies. The ability to measure biomarkers before, immediately after, and several hours post-injury offers valuable insights into how these biomarkers change over time, providing a better understanding of the injury response and pinpointing the source of biomarker release. Prior studies in murine models have explored the changes in NfL, phosphorylated tau 181 (pTau-181) and other biomarkers associated with BBB opening using focused ultrasound.^24,25^ However, no study has evaluated their trajectories in humans after MRgHIFU thalamotomy.

In this study, we compared changes in circulating levels of GFAP, NfL, amyloid beta species (Aβ40, Aβ42), and pTau-181 at three specific time points: before, 1 h after, and 48 h after MRgHIFU thalamotomy. To our knowledge, this is the first study to assess plasma biomarker changes in human subjects following MRgHIFU ablation. Our findings suggest that MRgHIFU thalamotomy leads to systemic biomarker changes across individuals and represents a powerful tool for acute brain injury biomarker discovery.

## Materials and methods

### Patient information

All patients voluntarily consented to participate in this study. We studied 30 patients who underwent MRgHIFU from January 2023 through March 2024 at a single academic institution (University of Texas Southwestern Medical Center, UTSW). Consent was obtained according to the Declaration of Helsinki^26^ and it was approved by the ethical committee at UTSW. Patients were consented to provide their demographic information, diagnosis, MRgHIFU treatment details and to undergo intravenous blood collection before and after the procedure. The patient data were collected in a prospective manner and included their basic demographics (gender, age and dexterity), disease diagnosis, duration and features of the tremor and prior medication history. The MRgHIFU procedure details were also collected, and included the treatment site, skull density ratio, number of sonications, mean and maximum temperature (T_max_, °C) reached, power (W) and energy (J) delivered, history of prior MRgHIFU, treatment response at 48 h, procedure side effects and MR imaging findings post-procedure.

### Blood processing

Blood collection was performed with purple-top K2 EDTA coated tubes immediately prior (baseline) to MRgHIFU and 1 h after MRgHIFU in the FUS suite at UTSW Medical Center. The 48 h (47.5–48.5 h) time point was collected at a follow-up outpatient appointment. A range of +/− 30 min on visit day was included to account for differences in time to arrival to the laboratory, and blood draw processing time. The samples were stored on ice for transport until further processing. The tubes were then spun down at 3900 rpm for 10 min at 4°C in a Beckman Coulter Allegra V-15R centrifuge. The plasma supernatant was then collected and aliquoted into O-ring screw cap 1 ml sterile tubes and stored in a −80°C freezer until further processing.

### Biomarker detection using single molecule arrays

The samples were then transported to the UTSW Microarray Core for neurological biomarker detection via the Quanterix Single Molecule Arrays (SiMoA) assay (Billerica, MA). The Quanterix Neurology 4-plex E (NfL, GFAP, Aβ40 and Aβ42), and SiMoA pTau-181 assays were used in this study. These assays employ a bead-based enzyme-linked immunosorbent assay technology, on which the immunocomplexes formed on single beads containing primary antibody and detection antibody, are isolated in arrays of 50-femtolitre reaction chambers, allowing for the detection of single protein molecules by fluorescence imaging.^27^ For the quanterix neurology 4-plex E assay the analytical lower limit of quantification (LLOQ) and limit of detection (LOD) of each biomarker were: GFAP LLOQ 2.89 pg/ml, GFAP LOD 0.441 pg/ml; NfL LLOQ 0.40 pg/ml, NfL LOD 0.090 pg/ml; Aβ40 LLOQ 1.02 pg/ml, Aβ40 LOD 0.384 pg/ml; Aβ42 LLOQ 0.378 pg/ml and Aβ42 LOD 0.136 pg/ml.^28^ For the SiMoA pTau-181 kit, the LLOQ was 2.0 pg/ml, LOD 0.62 pg/ml.^29^ All samples were run in parallel using technical duplicates. Samples from the same individual were run on the same plate to avoid intraindividual variability between different plates.

### MRI acquisition for magnetic resonance-guided high-intensity focused ultrasound

All patients underwent an MRI scan with diffusion tensor imaging using a Philips 3T MR Scanner (Philips, Best, The Netherlands). The MRI acquisition protocol was performed as previously published.^20^ An identical post-procedure MRI was completed in the same scanner as the pre-procedure scan to evaluate the MRgHIFU ablation lesion. This confirmed that the thalamotomy was successfully delivered on the patient-specific location based on the four tract tractography mapping.

### Statistical analysis

Statistical analyses were completed using GraphPad Prism. One-way analysis of variance (ANOVA) was used for comparison of biomarker levels across the different time points. Tukey’s multiple comparisons test was used for head-to-head comparisons between each time point. Two-way *t-*test was completed to evaluate differences between first-time versus second-time MRgHIFU thalamotomy patients and patients with and without cerebral small vessel disease (CSVD). A linear regression analysis was performed to determine the correlation between age and biomarker levels.

## Results

### Patient characteristics

Thirty subjects were included in this study. The average patient age was 72.1 years (SD 8.6). 22 males (73.3%) and 8 females (26.7%) underwent MRgHIFU thalamotomy. 8 patients (26.7%) received treatment with right-sided DRTT/VIM ablation, and 22 (73.3%) underwent left-sided ablation. 25 of the treated patients had a principal diagnosis of ET, 2 had a diagnosis of ET + tremor-dominant PD (ET + TDPD), and 2 were diagnosed with TDPD. One of the subjects had a concurrent diagnosis of right handwriting tremor and dystonia. Five of the patients had undergone a prior thalamotomy procedure on the contralateral side, and one had received prior treatment on the ipsilateral side with suboptimal response requiring re-treatment. All the treated patients had successful ablations without incident except one of them (Case #9), who was unable to complete the MRgHIFU ablation due to skull density considerations and nausea/vomiting during the procedure. In this patient, the treatment was terminated, and the post-operative MRI demonstrated a lack of an ablative lesion. Patient characteristics and the MRgHIFU treatment details are summarized in Table 1 and Supplementary Table S1, respectively.

**Table 1:** Summary of patient demographics, tremor diagnosis and characteristics

ID#	Gender	Age	Dexterity	Diagnosis	Duration	Characteristics
1	Male	74	R	ET	20 + y	Bilateral
2	Male	71	R	ET	15 y	Bilateral. L > R
3	Male	81	L	ET	35 y	Bilateral. L > R
4	Male	69	L	ET	20 + y	Bilateral
5	Male	68	R	ET	20 + y	Bilateral. L > R
6	Male	78	R	ET	30 + y	Bilateral. R > L
7	Male	68	R	R writing tremor + dystonia	10 + y	R writing tremor
8	Male	75	R	ET + dystonic head tremor	30 y	Bilateral. L > R
9	Female	69	R	ET	20 + y	Bilateral. Symmetric
10	Male	74	R	ET + orthostatic syncope	20 + y	Bilateral. Symmetric
11	Male	72	L	ET	50 + y	Bilateral. L > R
12	Female	39	R	ET	10 + y	Bilateral. L > R
13	Male	74	R	ET	12 y	Bilateral. L > R. Mild head tremor
14	Female	86	R	ET	35 y	Bilateral. Symmetric
15	Male	81	R	ET	10 y	Bilateral. L > R. Mild resting tremor
16	Male	85	R	Severe ET + TDPD	*ET:* 30 + y. *PD:* 5 y	*ET:* R > L action tremor *PD:* Resting tremor
17	Female	76	R	ET	50 y	Bilateral + axial + voice tremor. Head, chin, jaw tremor
18	Male	80	R	ET	16 y	Bilateral. R > L
19	Female	74	R	ET	30–40 y	Bilateral. R > L
20	Male	64	R	ET	30 y	Bilateral. L > R
21	Male	64	R	ET	20–30 y	Bilateral. R > L
22	Male	73	R	ET	10 y	Bilateral
23	Female	64	R	ET	10 + y	Bilateral L > R
24	Female	80	R	TDPD	20 y	Resting tremor
25	Male	73	R	ET	10 + y	Bilateral. Symmetric
26	Male	67	R	TDPD	5 y	Resting tremor
27	Male	68	R	ET	30 y	Bilateral. Symmetric
28	Male	72	R	ET + writing tremor	30 y	R writing tremor
29	Male	77	R	ET + TDPD	9 y	Bilateral action and resting tremor
30	Female	68	L	ET	14 y	L > R writing difficulty. Head and jaw tremor

ET, essential tremor; TDPD, tremor-dominant Parkinson’s disease; L, left; R, right; y, years.

### The levels of glial fibrillary acidic protein, neurofilament light chain, Aβ40 and Aβ42 increase 48 h after magnetic resonance-guided high-intensity focused ultrasound delivery

The mean concentration values, standard deviation and range for plasma GFAP, NfL, Aβ40, Aβ42 and pTau-181 pre-HIFU, 1 h post-HIFU and 48 h post-HIFU are listed in Table 2. Supplementary Table S2 includes the biomarker levels for each individual subject. Of note, the 1 h post-HIFU collection for Case #17 was discarded due to significant haemolysis.

**Table 2:** Quantitative measurement of biomarker levels assessed by the quanterix single molecule arrays (SiMoA) assay

Biomarker	Time point #1 concentration Baseline, pg/ml (SD) [range]	Time point #2 concentration 1 h post, pg/ml (SD) [range]	Time point #3 concentration 48 h post, pg/ml (SD) [range]
GFAP	114.1 (53.4) [44.4–220.7]	136.8 (83.0) [43.9–444.5]	492.3 (383.4) [119.5–1845.5]
NfL	21.9 (9.0) [9.8–45.8]	18.1 (7.5) [6.6–34.2]	27.9 (13.0) [10.2–61.6]
Aβ40	82.0 (16.6) [42.8–129.2]	77.9 (20.6) [29.3–155.1]	99.2 (23.6) [65–182.7]
Aβ42	4.9 (1.3) [1.1–8.2]	4.5 (1.2) [1.3–6.5]	5.8 (1.6) [1.8–10.5]
pTau-181	34.4 (28.0) [12.2–165.9]	25.2 (14.0) [13.7–73.7]	30.1 (13.1) [11.2–65.2]

**Figure 1:**
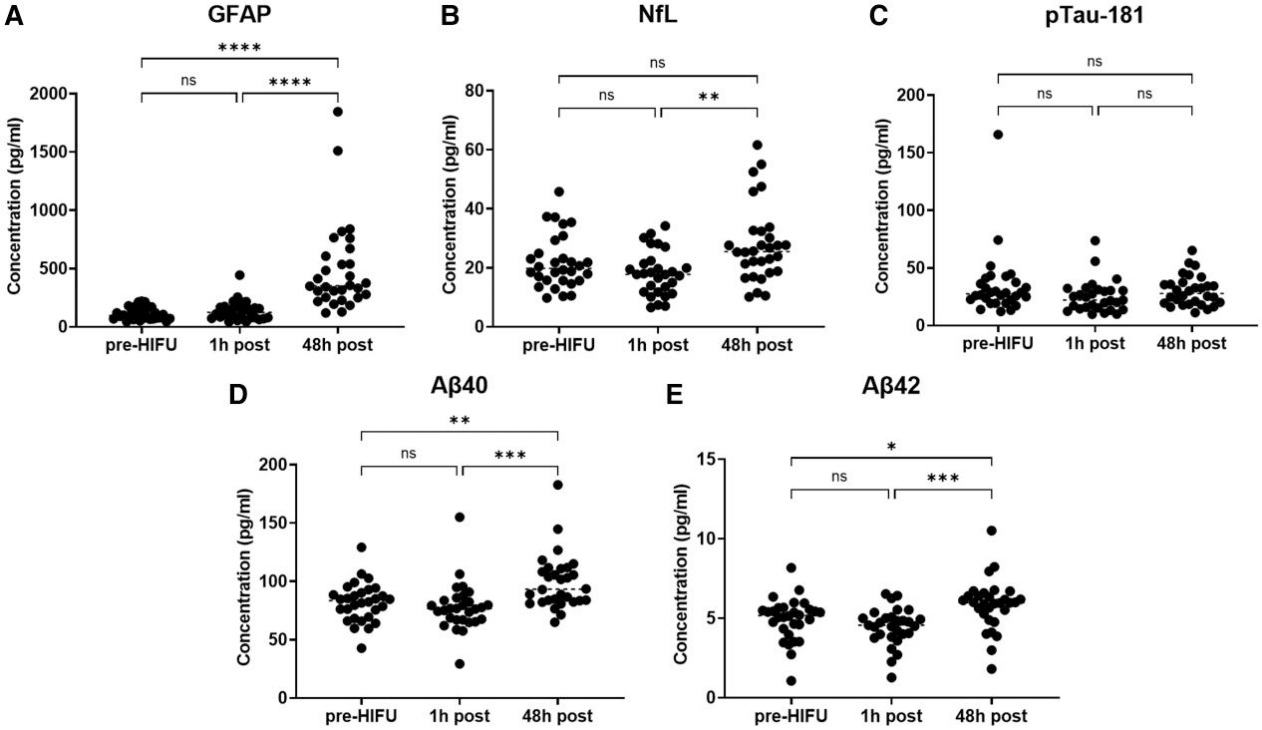
**Circulating plasma levels of GFAP, NfL, Aβ40, Aβ42, pTau-181 at baseline, 1 h post and 48 h post-HIFU.** The levels of GFAP (**A**), Aβ40 (**D**) and Aβ42 (**E**) were significantly elevated after 48 h when compared to the baseline and 1 h post-procedure levels. NfL levels (**B**) were significantly elevated after 48 h when compared to 1 h post-HIFU. No significant change was observed in the levels of pTau-181 (**C**). Each individual data point represents the concentration value of a biomarker for a particular individual. N = 30 individual measurements were included in the pre-HIFU and 48 h post-HIFU time points. N = 29 measurements were included in the 1 h post-HIFU time point (Case #17 was excluded due to gross haemolysis). A one-way ANOVA analysis was conducted with Tukey’s multiple comparisons test for head-to-head comparisons between each time point. (ns) *P*-value not significant; (*) *P*-value <0.05; (**) *P*-value <0.01; (***) *P*-value <0.001; (****) *P*-value <0.0001. Aβ40, amyloid beta 40; Aβ42, amyloid beta 42; ANOVA, analysis of variance; GFAP, glial fibrillary acidic protein; h, hour(s); HIFU, high-intensity focused ultrasound; NfL, neurofilament light chain; pg/ml, picograms/millilitre; pTau-181, phospho-tau 181.

**Figure 2:**
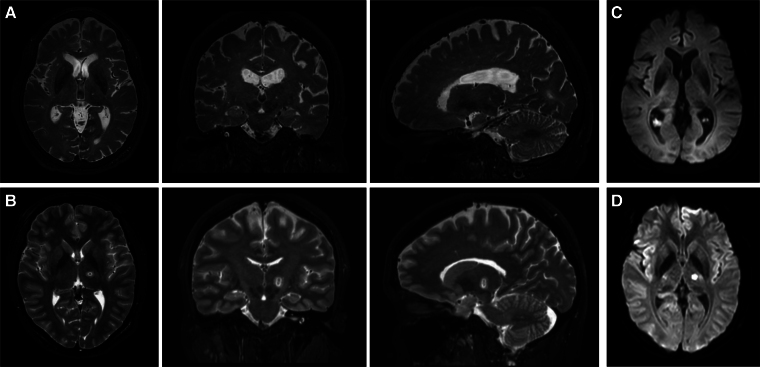
**MR imaging results for Case #9 and Case #12.** (**A** and **B**): The axial, coronal and sagittal views of the T2 weighted MR images are shown. No lesion was observed in the post-procedure MR for patient Case #9 (**A**). A typical MRgHIFU T2 weighted lesion is shown in (**B**) for comparison. (**C** and **D**): Axial view of diffusion-weighted imaging (DWI). No diffusion restriction was observed in Case #9 (**C**), in contrast to Case #12 (**D**). MR, magnetic resonance.

When the absolute plasma levels of these biomarkers were compared across the three time points (pre, 1 h post, and 48 h post-HIFU), we observed that plasma GFAP, Aβ40 and Aβ42 levels were significantly higher 48 h after MRgHIFU when compared to the baseline and 1-hour post-procedure level. In addition, NfL level was also significantly elevated at 48 h post-procedure when compared to 1 h post (Fig. 1). No significant differences were observed between the pre- and 1 h post-HIFU levels for any of the other biomarkers. Of all the biomarkers, GFAP levels exhibited the largest average increase at 48 h post-HIFU [Pre: 114.1 pg/ml (SD 53.4) versus 1 h-post: 136.8 pg/ml (SD 83.0) versus 48 h-post: 492.3 pg/ml (SD 383.4)]. Meanwhile, the levels of pTau-181 did not significantly change across any of the time points. In contrast to the rest of cases, Case #9 did not exhibit a statistically significant change in GFAP levels at 48 h. In this patient, the MRgHIFU ablation was terminated for treatment intolerance during the procedure. This prevented tissue ablation, which was verified on post-procedure MR (Fig. 2).

**Figure 3:**
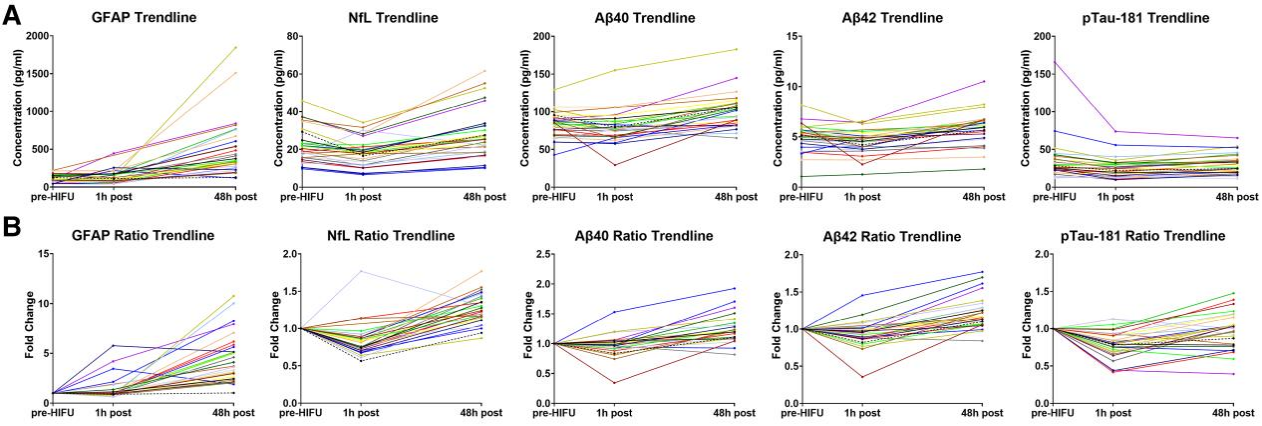
**Individual trajectories of plasma biomarkers in absolute values (A) and fold-change from baseline (B).** Case #9, in whom the procedure was terminated, and no lesion was generated, is identified with a ‘black dashed line’. GFAP levels were found to consistently increase at the 48-h time point across all the subjects, except for Case #9. The time points in the *x*-axis (pre, 1 h post, 48 h post) are not in linear scale. Aβ40, amyloid beta 40; Aβ42, amyloid beta 42; GFAP, glial fibrillary acidic protein; h, hour(s); HIFU, high-intensity focused ultrasound; NfL, neurofilament light chain; pg/ml, picograms/millilitre; pTau-181, phospho-tau 181.

Figure 3 depicts the detailed curves of biomarker trajectories for every individual in absolute values (**A**) and as fold-change with respect to baseline (pre-HIFU) (**B**). As shown, GFAP consistently increased across all the patients who completed MRgHIFU treatment except in Case #9. Of note, although the levels of GFAP 1 h post-HIFU did not increase significantly when compared to the pre-HIFU measurement (Fig. 1), when evaluating the individual GFAP trajectories, a notable increase was observed at 1 h post-HIFU in a few subjects. These included Case #3 (4.19-fold, 444.5 versus 106.1 pg/ml), Case #12 (5.76-fold, 255.7 versus 44.4 pg/ml), Case #21 (3.45-fold, 217.2 versus 63.0 pg/ml), Case #27 (1.85-fold, 127.3 versus 68.9 pg/ml) and Case #30 (2.14-fold, 157.2 versus 73.4 pg/ml). No unique characteristics were identified for these patients, except for Case #3 who had received bilateral thalamotomy.

Of note, we observed that the patient age correlated with the baseline levels of GFAP (R^2^ 0.37, *P* 0.0003), as well as the 48 h post-HIFU levels (R^2^ 0.14, *P* 0.0424). In addition, NfL levels also correlated with age at the three time points. This was not the case for Aβ40, Aβ42 and pTau-181 (Supplementary Fig. S1). When individuals were stratified based on the presence or CSVD, baseline GFAP levels were found to be elevated in those with CSVD compared to those without. However, there was a significant overlap in GFAP levels between the two groups. No differences were observed at any of the other time points or for any other biomarker (Supplementary Fig. S2).

### Glial fibrillary acidic protein serves as a sensitive and specific marker of magnetic resonance-guided high-intensity focused ultrasound ablation after 48 h

**Figure 4:**
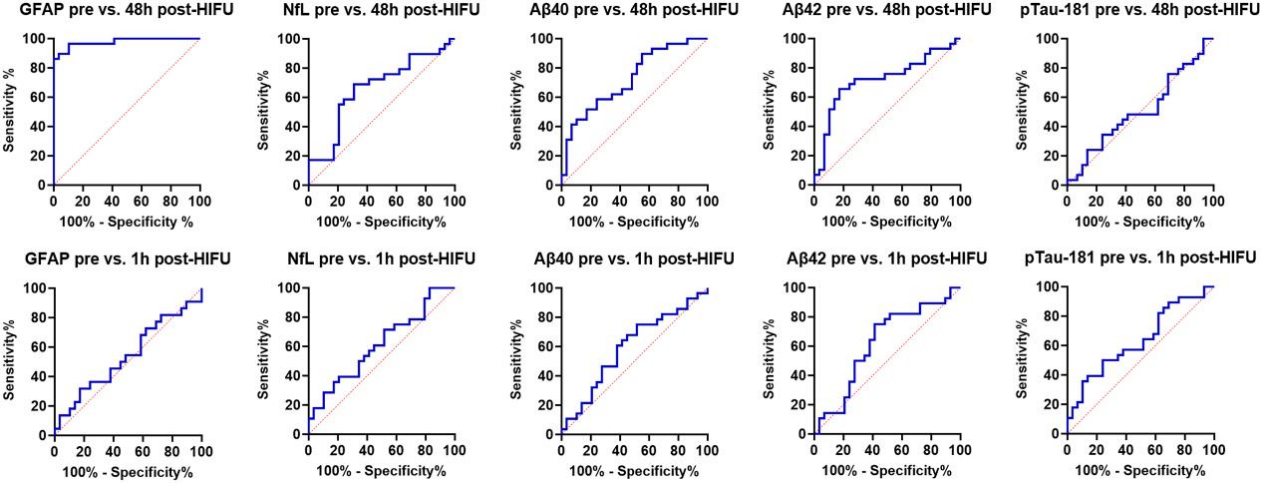
**ROC characteristics analysis for each plasma biomarker.** The results are plotted as 100%—Specificity % versus Sensitivity % for each biomarker. The levels of biomarkers at 48 h post-HIFU (top) or 1 h post-HIFU (bottom) were compared against the baseline pre-HIFU level. The GFAP pre- versus 48 h post-HIFU exhibited the best ROC fit. For this analysis, Case #9 was excluded due to the absence of lesion. N = 29 subjects were included in the pre-HIFU and 48 h post-HIFU groups. N = 28 subjects were included in the 1 h post-HIFU group as Case #17 was excluded due to gross haemolysis. Aβ40, amyloid beta 40; Aβ42, amyloid beta 42; GFAP, glial fibrillary acidic protein; h, hour(s); HIFU, high-intensity focused ultrasound; NfL, neurofilament light chain; pTau-181, phospho-tau 181.

We next tested the sensitivity and specificity of plasma biomarkers to determine the presence of MRgHIFU lesion. Case #9 was excluded given the absence of a thalamotomy lesion. We conducted a receiver operating characteristic (ROC) analysis by plotting the sensitivity against 1-specificity of our assay for each biomarker, with cases defined as post-HIFU samples (either 48 h post or 1 h post-HIFU), and the controls defined as the baseline pre-HIFU samples (Fig. 4). GFAP levels at 48 h post-HIFU versus pre-HIFU showed the best ROC fit, with an area under the ROC curve (AUC) of 0.9774 (95% CI 0.945 to 1, *P* < 0.0001) (Table 3). A GFAP cut-off value of 216.2 pg/ml exhibited a sensitivity of 89.7% (95% CI, 73.6% to 96.4%) and specificity of 96.6% (95% CI, 82.8% to 99.8%) to discriminate the presence of a thalamotomy lesion at 48 h post-HIFU versus absence of it (pre-HIFU). Using a 224.4 pg/ml cut-off value had a specificity of 100% (95% CI 88.3% to 100%), and a sensitivity of 86.2% (95% CI 69.4% to 94.5%). Lowering the cut-off to 112.8 ng/ml reached 100% sensitivity (95% CI 88.3% to 100%) but with a reduction in specificity to 58.6% (95% CI 40.7% to 74.5%). The sensitivity and specificity for each GFAP cut-off value at the 48 h post-HIFU time point versus pre-HIFU are shown in Supplementary Table S3. The rest of biomarkers exhibited a worse ROC fit than GFAP to discriminate presence versus absence of thalamotomy lesion (Fig. 4A). However, at the 48 h post-HIFU time point, the AUC for NfL, Aβ40 and Aβ42 was statistically significant (*P-*value <0.05) (Fig. 4B). No AUC reached statistical significance at the 1 h post-HIFU versus pre-HIFU time point in any of the biomarkers.

**Table 3:** Area under the curve, standard error, confidence interval and *P-*value for all biomarkers

Biomarker	Area under the curve (AUC)	Standard error (SE)	95% Confidence interval	*P*-value
48 h post-HIFU versus pre-HIFU				
GFAP	0.9774	0.017	0.95 to 1	**<0**.**0001**
NfL	0.6635	0.07	0.52 to 0.81	**0**.**0325**
Aβ40	0.7265	0.066	0.60 to 0.86	**0**.**0031**
Aβ42	0.7194	0.07	0.58 to 0.86	**0**.**0041**
pTau-181	0.5089	0.08	0.36 to 0.66	0.9071
1 h post-HIFU versus pre-HIFU				
GFAP	0.5392	0.084	0.38 to 0.70	0.6345
NfL	0.6121	0.075	0.47 to 0.76	0.1463
Aβ40	0.5948	0.076	0.45 to 0.74	0.2190
Aβ42	0.6232	0.076	0.47 to 0.77	0.1104
pTau-181	0.6355	0.074	0.49 to 0.78	0.0791

*P*-values < 0.05 are highlighted in bold.

### The increase in glial fibrillary acidic protein levels post-high-intensity focused ultrasound is higher in second-time magnetic resonance-guided high-intensity focused ultrasound patients at 48 h post-procedure

Next, we tested for differences in biomarker changes between those individuals with first-time unilateral MRgHIFU thalamotomy versus those undergoing a second ablation. Five subjects had a prior contralateral thalamotomy, and one had a prior ipsilateral treatment. The mean difference in treatment time between the first and second procedure was 14.2 months (SD 5.0) with the greatest interval being Case #18 (22 months), and the shortest being Case #22 (9 months). Case #9 was excluded from the analysis in this group, given the suboptimal MRgHIFU treatment.

**Figure 5:**
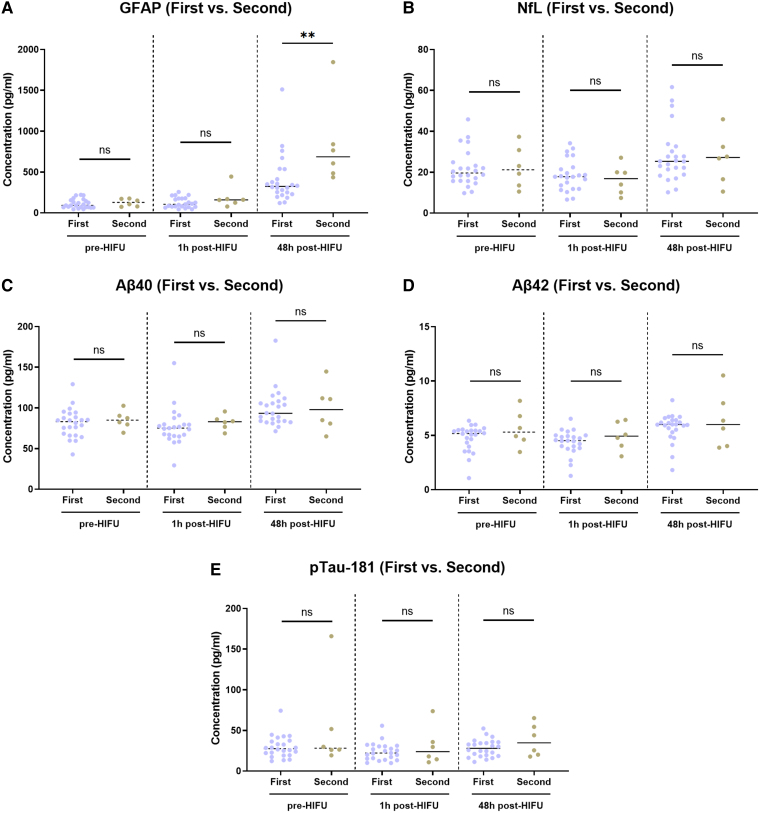
**Comparison analysis in absolute biomarker levels between first-time (N = 23) and second-time (N = 6) thalamotomy patients.** Biomarkers GFAP, NfL, Aβ40, Aβ42, pTau-181 levels were compared in first-time and second-time thalamotomy patients. Significantly higher levels of GFAP (**) were observed in the second-time thalamotomy patients at 48 h post-HIFU (**A**). No differences were observed for any of the other biomarkers (**B–E**). Each individual data point represents the concentration value of a biomarker for a particular individual. N = 23 individuals were included in the first-time thalamotomy group and N = 6 individuals were included in the second-time thalamotomy. A two-way *t*-test analysis was conducted between the two groups at each time point. *(*ns) *P*-value not significant; (*) *P*-value <0.05; (**) *P*-value <0.01; (***) *P*-value <0.001; (****) *P*-value <0.0001. Aβ40, amyloid beta 40; Aβ42, amyloid beta 42; GFAP, glial fibrillary acidic protein; h, hour(s); HIFU, high-intensity focused ultrasound; NfL, neurofilament light chain; pg/ml, picograms/millilitre; pTau-181, phospho-tau 181.

When the absolute concentrations of biomarkers were measured, baseline and 1 h post-HIFU levels were not significantly different between the two groups, but after 48 h, there was a significantly higher level of GFAP in those who underwent bilateral thalamotomy (Fig. 5). However, the interpretation of these findings is limited by the sample size differences between the groups, with 23 patients in the first-time versus 6 patients in the second-time group.

## Discussion

### Plasma glial fibrillary acidic protein as a consistent biomarker of thalamotomy injury

Our study found that MRgHIFU thalamotomy increased the plasma levels GFAP, NfL, Aβ40 and Aβ42 but not pTau-181 at 48 h after treatment delivery, with GFAP emerging as the most robust biomarker. GFAP levels increased in all individuals who developed a thalamotomy lesion, and notably, there was no increase in GFAP in Case #9, where the MRgHIFU delivery was insufficient to generate a lesion and the procedure was stopped. These findings suggest that GFAP is a reliable marker of focal brain injury after MRgHIFU thalamotomy.

Numerous studies have evaluated the use of GFAP as an acute marker of brain injury, particularly in acute stroke, as well as in TBI.^3^ In 2018, the FDA authorized the use of GFAP and ubiquitin carboxy-terminal hydrolase L1 (UCHL1) for clinical use in mild TBI.^3^ In the context of stroke, GFAP has been found to be elevated after both ischaemic^30^ and haemorrhagic injury (ICH).^31,32^ Some studies have also hypothesized using GFAP to differentiate between ICH and an ischaemic stroke in the acute setting.^16,33^ In ICH, there is a rapid GFAP increase due to the sudden BBB disruption, while in ischaemic stroke, a more gradual process is observed due to cytolysis and glial necrosis.^34^ The levels of GFAP in these stroke patients also correlated with severity and a history of a prior stroke.^33^ Other studies have also established GFAP as a possible prognostic marker of acute ischaemic stroke. GFAP levels prospectively correlated with clinical and rehabilitation outcomes.^5^ In another study, elevated GFAP on admission after ischaemic stroke, predicted poor functional outcomes during the 1-year follow-up.^30^ However, high variation has been observed in all the above studies regarding the GFAP cut-off values used for diagnosis of injury, which has limited the clinical applicability of this biomarker. Multiple reasons explain this variability, including differences in case versus control characteristics, timing of injury, population age and pre-existing comorbidities, lesion type and location.

### Establishment of a glial fibrillary acidic protein cut-off value for focal brain injury detection

One major limitation across all the above studies assessing brain injury biomarkers is the absence of a pre-event measurement that can allow for intraindividual comparison in the change in GFAP and other biomarker levels before and after injury. By using pre- and post-procedural plasma collection in our study, we overcame this limitation, which to our knowledge is unprecedented in brain injury biomarker research. Our study provides a unique opportunity to evaluate biomarker dynamics in a controlled and reproducible model of focal brain injury using MRgHIFU. The thalamic lesion generated is also highly consistent between individuals facilitating inter-individual comparison. Using this tool, we determined that GFAP levels assessed by SiMoA technology consistently increased across all patients 48 h after MRgHIFU, and that the use of a GFAP cut-off value of >216.2 pg/ml was highly sensitive and specific for detecting the presence of a lesion in these patients. In addition, for a small subset of patients, the levels of GFAP also increased at 1 h after MRgHIFU. Interestingly, GFAP did not elevate in the subject (Case #9) in whom the procedure was terminated prior to thermocoagulative necrosis and in whom the effect from the MRgHIFU procedure was transient. This suggests that GFAP may be specific for astroglial damage rather than subtotal transient effects. Our findings demonstrate that GFAP levels at 48 h post-MRgHIFU consistently increase across all patients with ablation lesions. Thus, GFAP serves as a robust marker of successful MRgHIFU thalamotomy.

### Glial fibrillary acidic protein as a surrogate of astrocytic damage

The pronounced and consistent elevation of GFAP observed within 48 h post-MRgHIFU likely reflects the presence of astroglial injury. GFAP is a cytoskeletal protein that is released into the bloodstream upon astrocytic damage.^3,7^ Astrocytes play a critical role in maintaining BBB integrity, and their injury can lead to the release of GFAP into the circulation.^7^ Studies have also demonstrated that astrocytes rapidly respond to neuronal injury by initiating a repair process forming a barrier around the lesion site to prevent further damage.^35^ Although reactive gliosis typically develops days to weeks after injury, the GFAP elevation seen in this study likely represent the initial phase of astrocytic injury and activation.^35^

Interestingly, although GFAP elevated in all subjects, there was a broad concentration range at the 48 h post-HIFU time point [GFAP 492.3 pg/ml (SD 383.4)], with a range of 119.5–1845 pg/ml. One potential explanation for these findings is that, although MRgHIFU generates a consistent ablative lesion in the thalamus the degree of BBB compromise may be different across different subjects.^22^ The variability in GFAP increase observed between individual cases in our study reflects the complex interplay of factors influencing biomarker release. These factors may include differences in the degree of BBB disruption, lesion size and individual astrocytic responses to injury. For example, cases with pronounced GFAP increases (e.g. Case #22) likely reflect more significant BBB disruption and astrocytic injury. Conversely, cases with lower (e.g. Case #13) or rapid but plateauing elevations (e.g. Case #12) may reflect different GFAP release kinetics or astrocytic activity.

In addition, as noted in prior studies, the patient age as well as pre-existing conditions may also impact the baseline biomarker levels and their trajectories.^36^ Indeed in our study there was a positive correlation between age and GFAP levels pre-HIFU and 48 h post-HIFU with the lowest baseline level seen in the youngest individual (Case #12). This was also the case for NfL, which showed the strongest correlation with age. In addition, comorbidities such as the presence of baseline CSVD can impact these levels. In our study, we observed a small but significant difference in baseline GFAP in those individuals with CSVD. Further research assessing the correlation between biomarkers and the severity of CSVD and other comorbidities can provide further insight into the baseline levels and trajectories of these biomarkers after injury.

### Potential clinical applications of plasma glial fibrillary acidic protein in brain injury

Our study provides further evidence for the use of GFAP as a marker of CNS thalamic injury and establishes a highly specific and sensitive cut-off value for this biomarker that could be translated to clinical use in other brain injuries such as acute stroke or TBI. Interestingly, we also observed that GFAP levels at 48 h were higher after second-time versus first-time thalamotomy. These results align with a prior study, which identified higher GFAP levels in acute stroke patients with a history of prior stroke.^33^ One potential explanation for this finding is that a history of prior thalamotomy might prime astrocytes, leading to an amplified response to subsequent injury. This phenomenon could reflect astrocytic sensitization or altered glial reactivity due to cumulative neural trauma.^37^ However, the interpretation of these results is limited by sample size differences between the groups.

Given the reliability of GFAP in detecting small thalamic injuries, it is plausible that GFAP could be effective for the detection of other types of injuries such as acute subcortical and cortical strokes.^38^ However, while our model allows for precise temporal and spatial control of thalamic injury, we acknowledge that the biomarker responses observed in this context may not directly translate to stroke or other types of acute brain insults. The MRgHIFU lesion represents a specific mechanism of focal brain injury, and further studies are needed to determine the applicability of these findings and the established cut-off in this study to other conditions, including ischaemic or haemorrhagic strokes, especially those in other locations beyond the thalamus.

While baseline GFAP values are unavailable in acute clinical settings, our study provides evidence that specific thresholds, such as the 216.2 pg/ml cut-off identified here, offer high sensitivity and specificity for detecting focal brain injury. Despite its sensitivity, there was indeed one case (Case #17) in which the baseline GFAP level (220.70 pg/ml) was higher than the established cut-off for injury. This patient had a notable history of chronic lacunar infarcts in the bilateral corona radiata, periventricular and white matter CSVD. Similarly, in three cases (Cases #4, #11, #13 and #21), the time point established did not reach the injury cut-off despite showing an increase from baseline level. The variability observed underscores the importance of further studies to elucidate the mechanisms driving differential GFAP release. Combining GFAP with other biomarkers or integrating dynamic changes over time may improve diagnostic accuracy.

Our findings contribute to the growing body of research on acute brain injury biomarkers, demonstrating the utility of MRgHIFU as a model system to study their dynamics. However, these results should be interpreted within the limitations of this specific injury mechanism. We suggest that future studies should explore using the established cut-off to determine whether GFAP can serve as a reliable detection marker in acute stroke and other brain injuries.

### Neurofilament light chain and Aβ40, Aβ42 also increase after thalamotomy injury

Plasma NfL, Aβ40, and Aβ42 also increased after 48 h post-HIFU. However, the changes were not as robust as GFAP and were not present across all subjects, with a significant overlap observed between the three time points (pre, 1 h post and 48 h post). As mentioned, the pronounced and consistent elevation of GFAP is suggestive of astrocytic injury and BBB disruption. In contrast, the more variable increases in NfL, Aβ40 and Aβ42 may reflect ongoing axonal injury or amyloid processing, which may be more dependent on time and lesion size. Plasma NfL quantified within the first 24 h of stroke has been proposed as a biomarker of stroke outcomes.^39^ However, a systematic review of the temporal trajectory of NfL in stroke determined that the levels of this biomarker significantly increased in the early subacute period after stroke, that is between 14 and 21 days after injury, when compared to the acute setting.^40^ Thus, assessment of time points beyond 48 h after MRgHIFU could potentially reveal a more consistent elevation of this marker. In addition, as mentioned above, plasma NfL positively correlated with age. Adjustment for age group may be necessary when using NfL as a biomarker of brain injury. Interestingly, we observed a slight decrease in NfL levels at the 1 h post-HIFU time point compared to pre-HIFU in some individuals, although this did not reach statistical significance. Localized acute effects of MRgHIFU, such as changes in regional cerebral perfusion or vascular occlusion could potentially explain this effect.^41^ However, this trend may also reflect baseline variability in NfL release kinetics unrelated to the procedure, or minor technical variability in sample handling and processing times despite standardized protocols. Further studies incorporating earlier post-procedure time points may help elucidate this trend.

### Plasma phosphorylated tau 181 does not change after thalamotomy

In contrast to the other biomarkers, we did not observe an increase in pTau-181 following thalamotomy. This lack of a robust pTau-181 response may be attributed to the lesion location, as tau-related pathophysiological changes are less pronounced in the thalamus compared to other regions, and may also take longer to manifest.^42^ Of note one of the cases (Case #3) exhibited a highly elevated pTau-181 level at baseline (pre-HIFU, 165.87 pg/ml), which downtrended at subsequent time points. The significance of this is unclear. While Case #3 had a history of prior thalamotomy and CSVD, this trajectory was not observed for other cases with a similar phenotype. Given this, we advise interpreting this with caution, and may have constituted an outlier laboratory error. While pTau-181 may not be useful as an acute brain injury biomarker, the use of focused ultrasound (FUS) liquid biopsy coupled with pTau species measurement has potential in neurodegenerative conditions. Targeting regions with classic tau pathology, such as the hippocampus, for FUS BBB opening in patients with cognitive impairment may enhance the diagnostic sensitivity of pTau-181 and other neurodegeneration biomarkers, as demonstrated in animal studies.^25^

### Study limitations

One limitation of our study is the inclusion of only three time points: pre-HIFU, 1 h post-HIFU and 48 h post-HIFU. Notably, the 48-h time point, where we consistently observed GFAP elevation, is not highly acute. Our study also noted an early GFAP elevation in some patients at 1 h post-HIFU. However, due to the limited time points used in our study, it is impossible to determine whether a consistent elevation in GFAP occurs earlier than 48 h across all patients or what the curve of biomarker elevation is across individuals, which could potentially guide acute clinical decisions in brain injury. Therefore, including additional time points between 1 and 48 h, as well as beyond 48 h, is crucial to gain a more comprehensive understanding of the trajectory and peak levels of biomarkers like GFAP and NfL post-HIFU. This would also provide critical insights that inform the development of biomarkers capable of guiding acute interventions in other brain injuries such as stroke.

It is also important to note that the population in our study encompassed an age range of 64–86 years old. Given this, including a broader age population and adjusting for comorbidities such as CSVD is necessary for further validation of the brain injury cut-off values set in this study for GFAP and other biomarkers.

## Supplementary Material

fcaf054_Supplementary_Data

## Data Availability

The authors confirm that the data supporting the findings of this study are available within the article and its Supplementary material.
