# Supplementary material for: Plasma glial fibrillary acidic protein as a biomarker of acute focal brain injury after high-intensity focused ultrasound thalamotomy
Source: Brain Commun. 2025 Mar 3;7(1):fcaf054. doi: 10.1093/braincomms/fcaf054 (PMC11997805; doi:10.1093/braincomms/fcaf054)
Supplement: fcaf054_Supplementary_Data [file fcaf054_Supplementary_Data.pdf]

Supplementary Table 1: Summary of MRgHIFU treatment characteristics

ID#	**Site**	**SDR**	**N° Sonic**	**T° target reached**	**Mean/ Max T°**	**Power, W**	**Energy, J**	**Prior HIFU?**	**Response at 48h**	**Side effects**	**MR findings**
1	L	0.61	9	No	56/59	1,044	25,666	No	Excellent (resolved)	Mild imbalance, lightheadedness	L DRTT ablation.
2	L	0.61	7	Yes	58/61	847	8,528	No	Excellent (resolved)	Mild leg imbalance, tongue numbness	L DRTT ablation. Advanced bilateral CSVD
3	L	0.60	8	Yes	55/57	1,200	24,618	Yes. R (1 y. 6 mo. prior)	Excellent (resolved)	Imbalance	L DRTT ablation. Prior R DRTT ablation. Periventricular and subcortical CSVD
4	R	0.56	5	Yes	56/58	996	11,015	No	Excellent (resolved)	Mild perioral numbness (resolved)	R DRTT ablation.
5	R	0.61	6	Yes	56/57	1,095	15,415	No	Excellent (resolved)	Subtle tongue numbness, mild imbalance	R DRTT ablation. Scattered periventricular and subcortical CSVD
6	L	0.46	6	Yes	58/62	1,099	15,415	No	Excellent (resolved)	Mild bottom lip numbness	L DRTT ablation. Mild CSVD
7	L	0.69	6	Yes	57/61	660	7,264	No	Excellent (resolved)	Mild imbalance	L DRTT ablation
8	R	0.69	7	Yes	57/59	896	8,987	Yes. L (10 mo. prior)	Excellent (resolved)	Mild imbalance, mild perioral numbness	R DRTT ablation. Prior L DRTT ablation
9	L	0.60	3	**No. Terminated**	45/46	300	3,002	No	Tremor 10% improved	Nausea, vomiting (terminated)	Small ablation zone in L thalamus with mild restricted effusion. Scattered foci of WM CSVD
10	L	0.46	4	No	49/50	1,000	9,118	No	Suboptimal response (50% improvement)	None	L DRTT ablation. Mild CSVD
11	R	0.55	6	Yes	55/58	798	8,773	No	Excellent (resolved)	None	R DRTT ablation. Mild CSVD, mild diffuse volume loss
12	L	0.80	7	Yes	55/60	750	8,250	No	Excellent (95% improved)	Slow walking for balance	L DRTT ablation
13	R	0.60	6	Yes	57/61	600	6040	No	Excellent (resolved)	Mild imbalance, improving	R DRTT ablation. Mild volume loss. Scattered CSVD
14	L	0.76	5	Yes	58/62	612	6080	No	Excellent (resolved)	Mild-moderate imbalance	L DRTT ablation. Mild volume loss. Mild periventricular and subcortical CSVD
15	L	0.62	7	Yes	56/59	850	9,413	No	Excellent (resolved)	Mild imbalance	L DRTT ablation. Scattered subcortical CSVD
16	L	0.49	4	Yes	50/53	556	7,822	No	Excellent (95% improved)	R lower lip numbness, mild imbalance	L DRTT ablation. Patchy subcortical CSVD
17	L	0.51	7	Yes	55/56	806	15,379	No	Excellent (resolved)	None	L DRTT ablation. Periventricular, WM CSVD. Chronic lacunar infarcts in bilateral corona radiata
18	R	0.43	7	Yes	50/52	1,097	28,577	Yes. L (1y. 10 mo. prior) -> 20% return	Excellent (90% improved)	None	R DRTT ablation. Prior L DRTT ablation
19	L	0.53	7	Yes	55/58	1,097	21,920	No	Excellent (resolved)	Mild imbalance	L DRTT ablation
20	L	0.59	6	Yes	57/62	995	11,946	No	Excellent (95% improved)	Slurred speech, mild R-sided imbalance	L DRTT ablation. Scattered periventricular and subcortical CSVD. Mild global cerebral volume loss
21	L	0.75	5	Yes	56/58	671	7,020	No	Excellent (95% improved)	Mild imbalance	L DRTT ablation
22	L	0.54	6	Yes	56/60	550	9,204	Yes. R (9 mo. prior) -> 5% tremor return	Excellent (resolved)	Mild bottom lip numbness	L DRTT ablation. Scattered mild CSVD
23	R	0.48	8	Yes	56/59	726	17,425	Yes. L (1 y. prior)	Excellent (resolved)	Very mild speech change	R DRTT ablation. Prior L DRTT ablation
24	L	0.44	9	Yes	57/61	897	12,551	No	Excellent (95% improved)	Mild-moderate imbalance, speech change	L DRTT ablation. Scattered periventricular, subcortical, splenium of corpus callosum CSVD
25	L	0.65	7	Yes	56/59	804	11,256	No	Excellent (95% improved)	None	L DRTT ablation
26	L	0.55	7	Yes	56/61	954	14,310	No	Excellent (resolved)	Imbalance, slowed gait	L DRTT ablation
27	L	0.57	7	Yes	54/57	799	10,393	No	Excellent (resolved)	Mild imbalance	L DRTT ablation
28	L	0.60	5	Yes	57/60	797	8,810	No	Excellent (resolved)	Mild numbness, dysgeusia	L DRTT ablation. Mild volume loss
29	R	0.55	5	Yes	57/60	894	12,572	No	Excellent (95% improved)	L perioral numbness	R DRTT ablation. Periventricular CSVD
30	R	0.59	5	Yes	57/60	550	13,200	Yes. Right (1 y. 2 mo. prior) -> Suboptimal	Excellent. Mild kinetic tremor, dystonic posture	L perioral numbness	R DRTT ablation. Bilateral CSVD

Abbreviations: CSVD, Cerebral Small Vessel Disease; DRTT, Dentatorubrothalamic tract; h, Hours; J, Joules; L, Left; Max, Maximum; mo., Months; MR, Magnetic Resonance; N° Sonic, Number of Sonications; R, Right; SDR, Skull Density Ratio; T°, Temperature; W, Watts; WM, White Matter

Supplementary Table 2: Individual patient GFAP, NfL, Aβ40, Aβ42, and pTau-181 biomarker levels at baseline, 1h post-MRgHIFU, and 48h post-MRgHIFU

**ID #**	**GFAP (pg/ml)**			**NfL (pg/ml)**			**Aβ40 (pg/ml)**			**Aβ42 (pg/ml)**			**pTau-181 (pg/ml)**		
	Pre	***1h Post***	***48 h Post***	***Pre***	***1h Post***	***48 h Post***	***Pre***	***1h Post***	***48 h Post***	***Pre***	***1h Post***	***48 h Post***	***Pre***	***1h Post***	***48 h Post***
1	101.31	97.22	308.59	45.82	34.18	52.51	129.20	155.05	182.70	5.95	6.53	8.24	36.31	30.42	32.69
2	143.37	124.62	331.14	35.45	31.61	55.07	92.88	75.12	111.71	5.39	4.73	6.59	41.11	32.81	42.14
3	106.07	444.47	839.77	37.31	27.08	45.87	90.27	95.65	144.81	6.77	6.42	10.51	165.87	73.67	65.17
4	65.51	73.02	217.55	15.79	13.54	16.19	81.54	73.65	83.90	5.54	4.84	6.19	12.17	13.72	11.23
5	70.67	79.91	355.80	21.80	18.05	27.72	94.36	94.92	115.09	5.06	5.00	5.92	28.80	21.62	30.09
6	45.02	44.07	253.37	21.96	19.51	32.64	59.54	58.53	101.67	3.96	3.99	6.40	32.98	26.79	34.37
7	52.45	54.58	307.29	18.77	21.40	25.33	76.21	67.00	88.81	5.20	4.47	5.52	22.17	20.09	30.79
8	175.41	160.63	435.25	19.25	13.95	27.61	79.63	76.74	65.00	4.60	4.05	3.87	26.35	17.95	25.47
9	124.41	110.95	128.18	29.32	16.55	27.57	95.32	79.47	105.57	5.14	4.21	5.66	28.38	22.17	24.68
10	154.66	175.24	375.72	24.86	18.43	33.76	88.47	90.90	105.52	4.76	4.56	5.96	24.96	19.76	19.45
11	90.82	76.96	185.14	15.64	17.76	18.28	87.54	64.89	108.18	5.49	4.01	6.71	37.45	24.65	35.86
12	44.42	255.67	228.09	10.34	7.04	11.49	59.80	57.62	76.59	4.33	3.76	4.89	22.72	9.93	16.24
13	90.94	86.06	194.83	14.57	10.29	16.94	76.18	79.80	82.15	5.07	4.93	5.28	23.85	15.25	24.55
14	180.24	174.27	535.40	20.36	17.73	25.27	85.36	29.33	88.95	6.34	2.26	6.71	25.63	25.27	34.18
15	131.39	178.78	539.48	37.16	28.16	47.51	68.29	67.02	103.06	1.06	1.27	1.80	42.73	32.27	32.50
16	214.13	213.56	1511.03	34.87	28.30	61.64	106.29	106.24	126.74	2.72	2.70	2.99	32.52	27.43	37.48
17	220.70	-*	818.79	17.95	-*	21.37	99.06	-*	118.20	5.44	-*	6.17	26.59	-*	21.09
18	150.25	168.56	765.27	23.04	19.95	32.38	82.37	83.56	110.88	4.92	4.77	6.35	29.77	29.65	43.96
19	166.87	163.14	349.76	21.82	18.66	25.63	84.70	77.66	103.81	5.65	4.50	6.00	28.75	30.32	35.51
20	69.37	43.92	250.38	18.42	11.22	22.17	84.71	76.76	92.97	4.65	4.45	6.00	13.29	12.44	15.93
21	62.96	217.23	119.52	9.76	6.57	10.15	42.78	65.45	82.35	3.33	4.85	5.90	74.26	55.77	52.26
22	171.48	124.53	1845.50	30.82	19.72	26.77	102.66	85.89	111.85	8.17	6.26	7.95	51.67	35.71	54.31
23	78.46	77.60	484.48	13.50	10.12	16.54	69.44	68.72	84.87	3.47	3.07	4.01	25.94	10.81	17.77
24	214.86	219.02	671.58	18.48	14.98	27.64	65.93	62.10	80.99	5.36	5.00	6.77	19.37	13.24	18.47
25	68.47	63.02	342.23	23.15	22.39	30.18	85.03	87.52	93.59	5.98	5.53	6.40	43.32	31.14	25.74
26	75.83	70.55	760.14	12.82	11.83	18.82	66.09	67.61	71.35	5.37	5.54	5.98	44.60	40.37	45.64
27	68.88	127.27	278.88	17.06	30.17	22.17	64.06	76.22	83.54	3.53	3.82	4.76	13.95	15.79	13.94
28	89.95	79.59	413.02	15.67	11.78	23.86	75.53	74.37	83.85	3.52	3.59	4.11	17.22	9.76	17.94
29	119.80	104.74	315.90	20.69	17.34	23.00	78.68	79.35	86.54	5.44	5.36	6.14	19.11	17.16	22.72
30	73.41	157.16	606.06	10.52	7.39	10.52	87.46	82.38	80.73	5.68	5.07	5.63	19.32	14.48	20.14

*The 1h post-HIFU sample from Case #17 was discarded due to gross hemolysis

**Supplementary Table 3:** Sensitivity and specificity for each GFAP cutoff to discriminate presence (48h post) vs. absence of thalamotomy lesion				
**Cutoff Value**	**Sensitivity%**	**95% Confidence Interval**	**Specificity%**	**95% Confidence Interval**
> 44.72	100	88.30% to 100.0%	3.448	0.1769% to 17.18%
> 48.74	100	88.30% to 100.0%	6.897	1.225% to 21.96%
> 57.71	100	88.30% to 100.0%	10.34	3.581% to 26.39%
> 64.23	100	88.30% to 100.0%	13.79	5.497% to 30.56%
> 66.99	100	88.30% to 100.0%	17.24	7.598% to 34.55%
> 68.68	100	88.30% to 100.0%	20.69	9.846% to 38.39%
> 69.13	100	88.30% to 100.0%	24.14	12.22% to 42.11%
> 70.02	100	88.30% to 100.0%	27.59	14.70% to 45.72%
> 72.04	100	88.30% to 100.0%	31.03	17.28% to 49.23%
> 74.62	100	88.30% to 100.0%	34.48	19.94% to 52.65%
> 77.15	100	88.30% to 100.0%	37.93	22.69% to 56.00%
> 84.20	100	88.30% to 100.0%	41.38	25.51% to 59.26%
> 90.39	100	88.30% to 100.0%	44.83	28.41% to 62.45%
> 90.88	100	88.30% to 100.0%	48.28	31.39% to 65.57%
> 96.13	100	88.30% to 100.0%	51.72	34.43% to 68.61%
> 103.7	100	88.30% to 100.0%	55.17	37.55% to 71.59%
> 112.8	100	88.30% to 100.0%	58.62	40.74% to 74.49%
> 119.7	96.55	82.82% to 99.82%	58.62	40.74% to 74.49%
> 125.6	96.55	82.82% to 99.82%	62.07	44.00% to 77.31%
> 137.4	96.55	82.82% to 99.82%	65.52	47.35% to 80.06%
> 146.8	96.55	82.82% to 99.82%	68.97	50.77% to 82.72%
> 152.5	96.55	82.82% to 99.82%	72.41	54.28% to 85.30%
> 160.8	96.55	82.82% to 99.82%	75.86	57.89% to 87.78%
> 169.2	96.55	82.82% to 99.82%	79.31	61.61% to 90.15%
> 173.4	96.55	82.82% to 99.82%	82.76	65.45% to 92.40%
> 177.8	96.55	82.82% to 99.82%	86.21	69.44% to 94.50%
> 182.7	96.55	82.82% to 99.82%	89.66	73.61% to 96.42%
> 190.0	93.1	78.04% to 98.77%	89.66	73.61% to 96.42%
> 204.5	89.66	73.61% to 96.42%	89.66	73.61% to 96.42%
> 214.5	89.66	73.61% to 96.42%	93.1	78.04% to 98.77%
> 216.2	89.66	73.61% to 96.42%	96.55	82.82% to 99.82%
> 219.1	86.21	69.44% to 94.50%	96.55	82.82% to 99.82%
> 224.4	86.21	69.44% to 94.50%	100	88.30% to 100.0%
> 239.2	82.76	65.45% to 92.40%	100	88.30% to 100.0%
> 251.9	79.31	61.61% to 90.15%	100	88.30% to 100.0%
> 266.1	75.86	57.89% to 87.78%	100	88.30% to 100.0%
> 293.1	72.41	54.28% to 85.30%	100	88.30% to 100.0%
> 307.9	68.97	50.77% to 82.72%	100	88.30% to 100.0%
> 312.2	65.52	47.35% to 80.06%	100	88.30% to 100.0%
> 323.5	62.07	44.00% to 77.31%	100	88.30% to 100.0%
> 336.7	58.62	40.74% to 74.49%	100	88.30% to 100.0%
> 346.0	55.17	37.55% to 71.59%	100	88.30% to 100.0%
> 352.8	51.72	34.43% to 68.61%	100	88.30% to 100.0%
> 365.8	48.28	31.39% to 65.57%	100	88.30% to 100.0%
> 394.4	44.83	28.41% to 62.45%	100	88.30% to 100.0%
> 424.1	41.38	25.51% to 59.26%	100	88.30% to 100.0%
> 459.9	37.93	22.69% to 56.00%	100	88.30% to 100.0%
> 509.9	34.48	19.94% to 52.65%	100	88.30% to 100.0%
> 537.4	31.03	17.28% to 49.23%	100	88.30% to 100.0%
> 572.8	27.59	14.70% to 45.72%	100	88.30% to 100.0%
> 638.8	24.14	12.22% to 42.11%	100	88.30% to 100.0%
> 715.9	20.69	9.846% to 38.39%	100	88.30% to 100.0%
> 762.7	17.24	7.598% to 34.55%	100	88.30% to 100.0%
> 792.0	13.79	5.497% to 30.56%	100	88.30% to 100.0%
> 829.3	10.34	3.581% to 26.39%	100	88.30% to 100.0%
> 1175	6.897	1.225% to 21.96%	100	88.30% to 100.0%
> 1678	3.448	0.1769% to 17.18%	100	88.30% to 100.0%

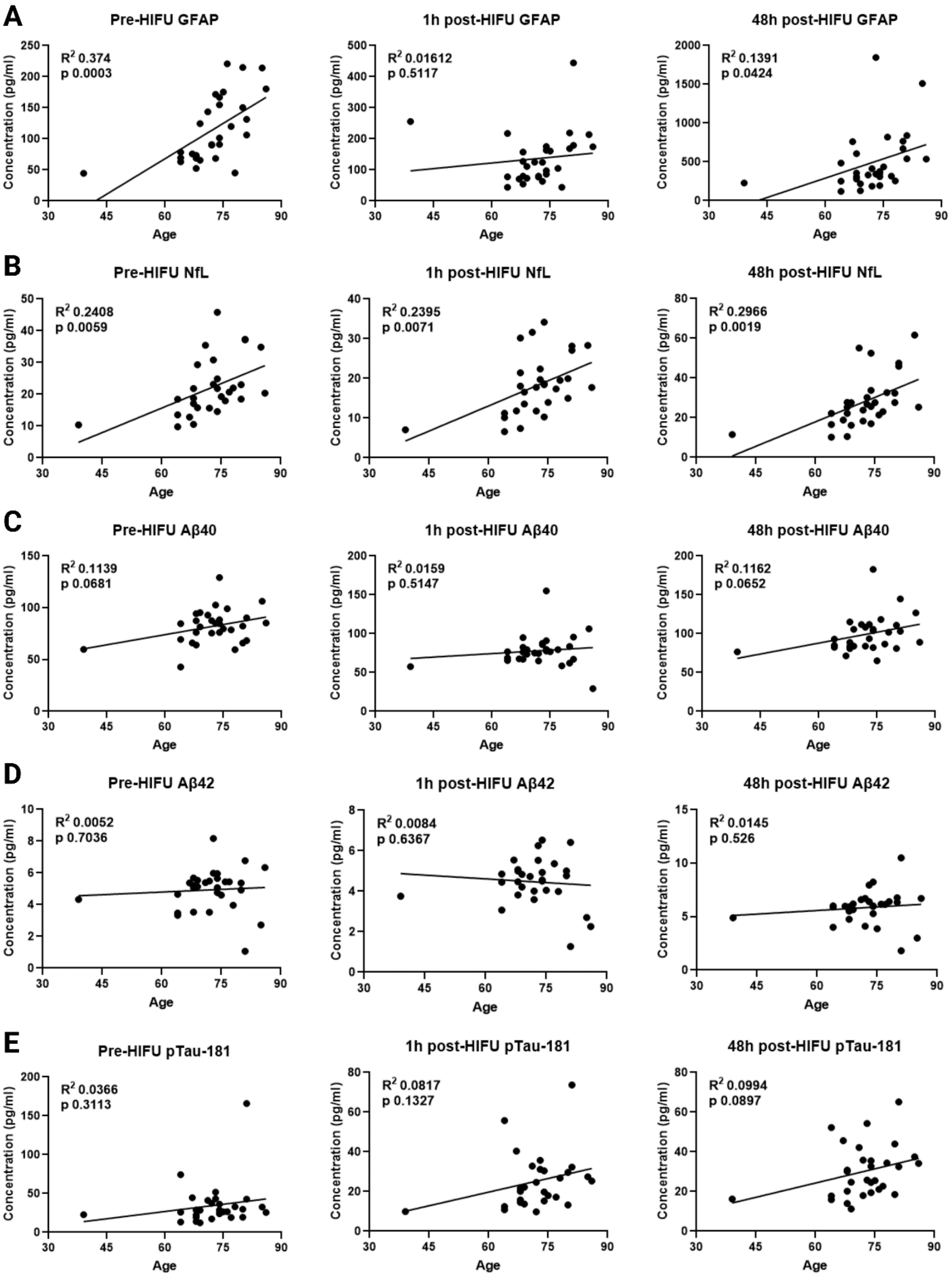
A
Pre-HIFU GFAP
R2 0.374
p 0.0003
1h post-HIFU GFAP
R2 0.01612
p 0.5117
48h post-HIFU GFAP
R2 0.1391
p 0.0424
B
Pre-HIFU NfL
R2 0.2408
p 0.0059
1h post-HIFU NfL
R2 0.2395
p 0.0071
48h post-HIFU NfL
R2 0.2966
p 0.0019
C
Pre-HIFU Aβ40
R2 0.1139
p 0.0681
1h post-HIFU Aβ40
R2 0.0159
p 0.5147
48h post-HIFU Aβ40
R2 0.1162
p 0.0652
D
Pre-HIFU Aβ42
R2 0.0052
p 0.7036
1h post-HIFU Aβ42
R2 0.0084
p 0.6367
48h post-HIFU Aβ42
R2 0.0145
p 0.526
E
Pre-HIFU pTau-181
R2 0.0366
p 0.3113
1h post-HIFU pTau-181
R2 0.0817
p 0.1327
48h post-HIFU pTau-181
R2 0.0994
p 0.0897
Concentration (pg/ml)
Age

Supplementary Figure 1: Correlation between patient age and plasma GFAP, NfL, Aβ40, Aβ42, pTau-181. (A): The pre-HIFU (R2 0.37, p 0.0003) and 48 h post-HIFU (R2 0.14, p 0.0424) GFAP levels had a positive correlation with age. (B): The NfL levels correlated with age at the three time points (Pre-HIFU: R^2 0.24, p 0.0059; 1h post: R^2 0.24, p 0.0071; 48h post: R^2 0.30, p 0.0019). No correlation with age was observed for Aβ40 (C), Aβ42 (D), or pTau-181 (E). N=30 individual measurements were included in the pre-HIFU and 48h post-HIFU time points. N=29 measurements were included in the 1h post-HIFU time point. A linear regression analysis was conducted between age and every biomarker at each time point. *Abbreviations:* Aβ40, Amyloid beta 40; Aβ42, Amyloid beta 42; GFAP, glial fibrillary acidic protein; h, hour(s); HIFU, High-intensity focused ultrasound; NfL, neurofilament light chain; p, p-value; pg/ml, picograms/milliliter; pTau-181, phospho-tau 181; R^2, coefficient of determination.

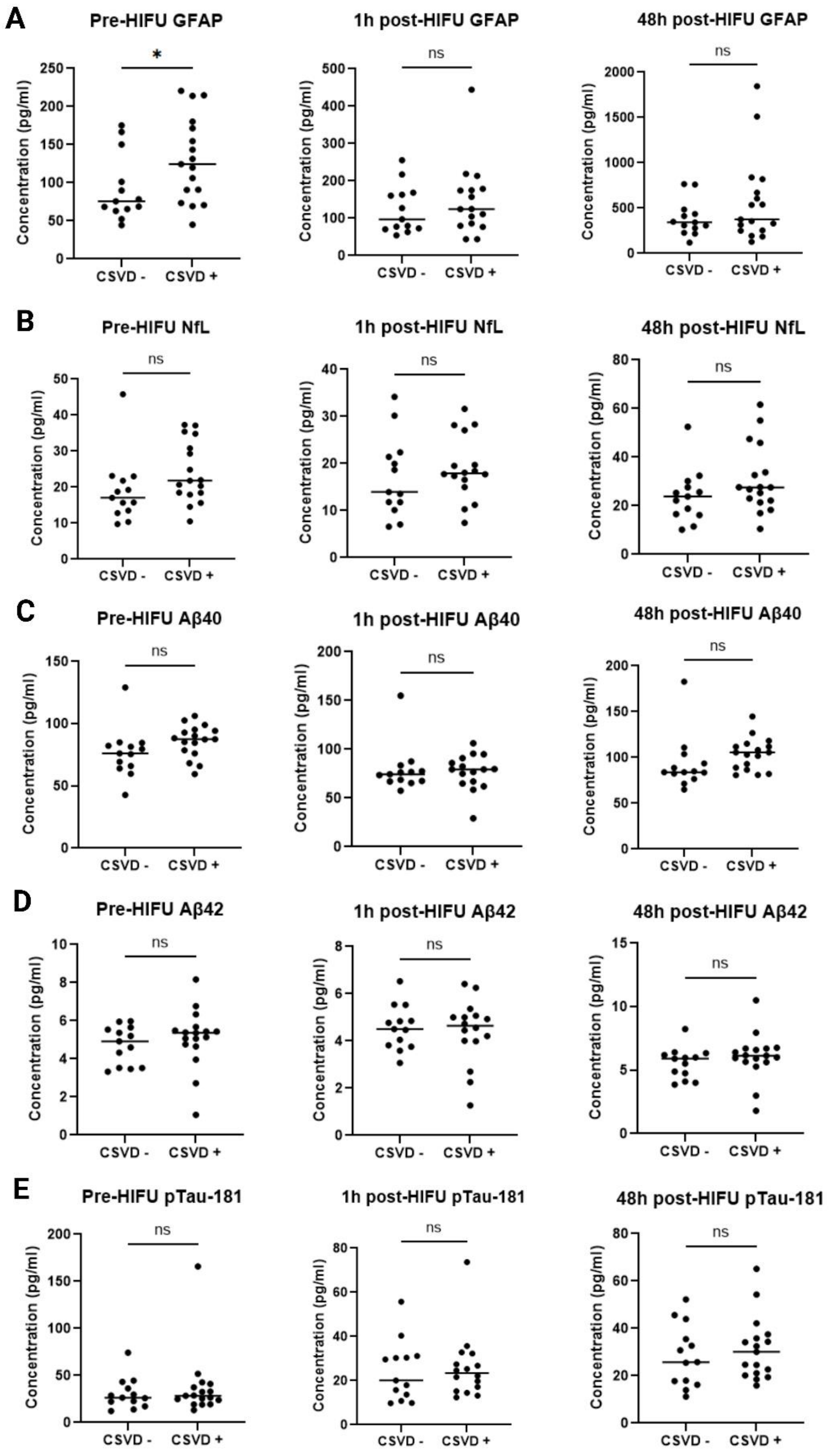

A
Pre-HIFU GFAP
1h post-HIFU GFAP
48h post-HIFU GFAP
B
Pre-HIFU NfL
1h post-HIFU NfL
48h post-HIFU NfL
C
Pre-HIFU Aβ40
1h post-HIFU Aβ40
48h post-HIFU Aβ40
D
Pre-HIFU Aβ42
1h post-HIFU Aβ42
48h post-HIFU Aβ42
E
Pre-HIFU pTau-181
1h post-HIFU pTau-181
48h post-HIFU pTau-181
Concentration (pg/ml)
CSVD -
CSVD +
*
ns

Supplementary Figure 2: Plasma biomarker levels in patients with and without cerebral small vessel disease (CSVD). (A): The baseline (pre-HIFU) GFAP levels were found to be higher (*) in patients with CSVD+ as assessed by MR imaging when compare to those without (CSVD -). No differences between groups were observed in any of the other time points or in any of the other biomarkers (B-E). N=17 individuals were included in the CSVD+ group at pre-HIFU and 48h post-HIFU. N=16 individuals were included in the CSVD+ group at 1h post-HIFU (Case #17 was excluded due to sample gross hemolysis). N=13 individuals were included in the CSVD- group. A two-way t test was conducted for comparisons between groups. (ns) p-value not significant; (*) p-value 0.05; (**) p-value 0.01; (***) p-value 0.001; (****) p-value 0.0001. *Abbreviations:* Aβ40, Amyloid beta 40; Aβ42, Amyloid beta 42; CSVD, cerebral small vessel disease; GFAP, glial fibrillary acidic protein; h, hour(s); HIFU, High-intensity focused ultrasound; NfL, neurofilament light chain; pg/ml, picograms/milliliter; pTau-181, phospho-tau 181.
